# Repeat Prostate Biopsy Strategies after Initial Negative Biopsy: Meta-Regression Comparing Cancer Detection of Transperineal, Transrectal Saturation and MRI Guided Biopsy

**DOI:** 10.1371/journal.pone.0057480

**Published:** 2013-02-27

**Authors:** Adam W. Nelson, Rebecca C. Harvey, Richard A. Parker, Christof Kastner, Andrew Doble, Vincent J. Gnanapragasam

**Affiliations:** 1 Department of Urology, Addenbrooke’s Hospital, Cambridge, United Kingdom; 2 Centre for Applied Medical Statistics, University of Cambridge, Cambridge, United Kingdom; 3 Translational Prostate Cancer Group, Hutchison/MRC Research centre, University of Cambridge, Cambridge, United Kingdom; The Chinese University of Hong Kong, Hong Kong

## Abstract

**Introduction:**

There is no consensus on how to investigate men with negative transrectal ultrasound guided prostate biopsy (TRUS-B) but ongoing suspicion of cancer. Three strategies used are transperineal (TP-B), transrectal saturation (TS-B) and MRI-guided biopsy (MRI-B). We compared cancer yields of these strategies.

**Methods:**

Papers were identified by search of Pubmed, Embase and Ovid Medline. Included studies investigated biopsy diagnostic yield in men with at least one negative TRUS-B and ongoing suspicion of prostate cancer. Data including age, PSA, number of previous biopsy episodes, number of cores at re-biopsy, cancer yield, and Gleason score of detected cancers were extracted. Meta-regression analyses were used to analyse the data.

**Results:**

Forty-six studies were included; 12 of TS-B, 14 of TP-B, and 20 of MRI-B, representing 4,657 patients. Mean patient age, PSA and number of previous biopsy episodes were similar between the strategies. The mean number of biopsy cores obtained by TP-B and TS-B were greater than MRI-B. Cancer detection rates were 30·0%, 36·8%, and 37·6% for TS-B, TP-B, and MRI-B respectively. Meta-regression analysis showed that MRI-B had significantly higher cancer detection than TS-B. There were no significant differences however between MRI-B and TP-B, or TP-B and TS-B. In a sensitivity analysis incorporating number of previous biopsy episodes (36 studies) the difference between MRI-B and TP-B was not maintained resulting in no significant difference in cancer detection between the groups. There were no significant differences in median Gleason scores detected comparing the three strategies.

**Conclusions:**

In the re-biopsy setting, it is unclear which strategy offers the highest cancer detection rate. MRI-B may potentially detect more prostate cancers than other modalities and can achieve this with fewer biopsy cores. However, well–designed prospective studies with standardised outcome measures are needed to accurately compare modalities and define an optimum re-biopsy approach.

## Introduction

Prostate cancer is the most common male cancer [1]. The vast majority of cancers are diagnosed from a transrectal ultrasound guided biopsy of the prostate (TRUS-B) following the finding of a raised PSA or abnormal digital rectal examination. The diagnostic yield of a first transrectal ultrasound guided biopsy is commonly 40–50% [2]. This represents a significantly high detection rate from a primary diagnostic intervention. It is however known that first-line prostate biopsy protocols such as traditional TRUS-B, even when used as an extended biopsy protocol of 12 cores will miss about 30% of prostate cancers [3]. Cancer detection rates in repeat TRUS-B range from 18% [4] to 32% [5]. Cancer will still be detected after multiple repeat TRUS-Bs, though the cancer detection rate falls with each repeat biopsy episode [6]. Even so the standard TRUS-B only allows limited access to the prostate and from the same anatomical approach, typically resulting in undersampling of the prostatic apex and anterior region of the gland [7].

A number of different techniques for prostate re-biopsy have been developed and tested in an attempt to improve cancer detection rates following an initial negative biopsy. These include saturation biopsy approaches (either transperineal or transrectal) or image guided (typically Magnetic Resonance Imaging) biopsies. In the initial biopsy setting it has been shown that the use of saturation biopsy techniques, which obtain greater number of prostate cores, either by transrectal or transperineal routes have no advantage over standard TRUS-B with respect to cancer detection rate [8], [9]. In the repeat biopsy setting however, it is known that the use of saturation biopsy detects more cancer than TRUS-B alone [3], [10]. MRI is well established as the staging modality of choice for prostate cancer; particularly in the assessment of extracapsular extension [11] but the use of MRI guided prostate biopsy (MRI-B) for diagnosis is still relatively new. A number of different techniques have been described for both the imaging of the prostate and the approach used to obtain the biopsy specimen. The use of T1 and T2-weighted MRI, [12] diffusion weighted MRI (MRI-DW), [13] MR spectroscopy (MRSI), [14] dynamic contrast enhanced MRI (DCE-MRI), [15] and various combinations of the above in multi-parametric imaging protocols [16]–[18] have all been described for the identification of prostatic lesions for targeted biopsy. The biopsy specimen can then be obtained by using transrectal, [12] transperineal, [19] or transgluteal approaches [20]. In the initial biopsy setting MRI guided biopsies have been shown to have a cancer detection rate of 54% [21]. A recent systematic review has reported that although requiring fewer cores MRI guided biopsies had similar initial detection rates compared to standard TRUS-B [22]. Thus in the initial biopsy setting neither transperineal, transrectal saturation biopsy, or image guidance have been shown to increase the yield of cancers nor indeed improve detection of clinically significant tumours [8], [9], [22].

What remains unclear from the existing literature is which repeat biopsy strategy offers the highest cancer detection rate in patients with a negative TRUS-B but ongoing suspicion of prostate cancer [23]. This group is arguably the one most in need of an optimal and unified strategy and where there is a justifiable need for resource intensive alternative to standard TRUS-B. In this study, we asked what the comparative cancer detection rates were between the three repeat biopsy strategies of transperineal biopsy (TP-B), transrectal saturation biopsy (TS-B), and MRI guided biopsy (MRI-B).

## Methods

### Search Criteria

A literature search of Pubmed, Embase and Ovid Medline databases was performed using the search terms ‘prostate’ and ‘biopsy’. Results were limited to the English language published since 1st January 1995 and up to January 2012. Three re-biopsy strategies were compared in this study: TP-B; multiple needle core prostate biopsy obtained through the perineum with or without the use of a brachytherapy grid template under transrectal ultrasound (TRUS) guidance; TS-B; multiple needle core prostate biopsy obtained transrectally under TRUS guidance and MRI-B; multiple needle core prostate biopsy obtained after the use of MRI to identify areas of the prostate suspicious for the presence of cancer. We did not distinguish between the various functional imaging modalities or biopsy approaches used in MRI-B for simplicity of analysis due to the diversity of techniques described in the current literature. The inclusion criteria for this study were papers investigating biopsy diagnostic yield in men who had one or more previous negative TRUS-B, who were undergoing one of the three repeat biopsy strategies outlined above for ongoing suspicion of prostate cancer, defined as elevated or rising PSA, abnormal digital rectal examination (DRE) of the prostate and/or previous high grade prostatic intraepithelial neoplasia (HGPIN) or atypical small acinar proliferation (ASAP) on previous prostate biopsy. Studies of men with known prostate cancer were excluded. In papers where both initial and repeat biopsies were being studied, only data relating to patients undergoing the repeat biopsy procedure were analysed.

### Data Analysis

**Figure 1:**
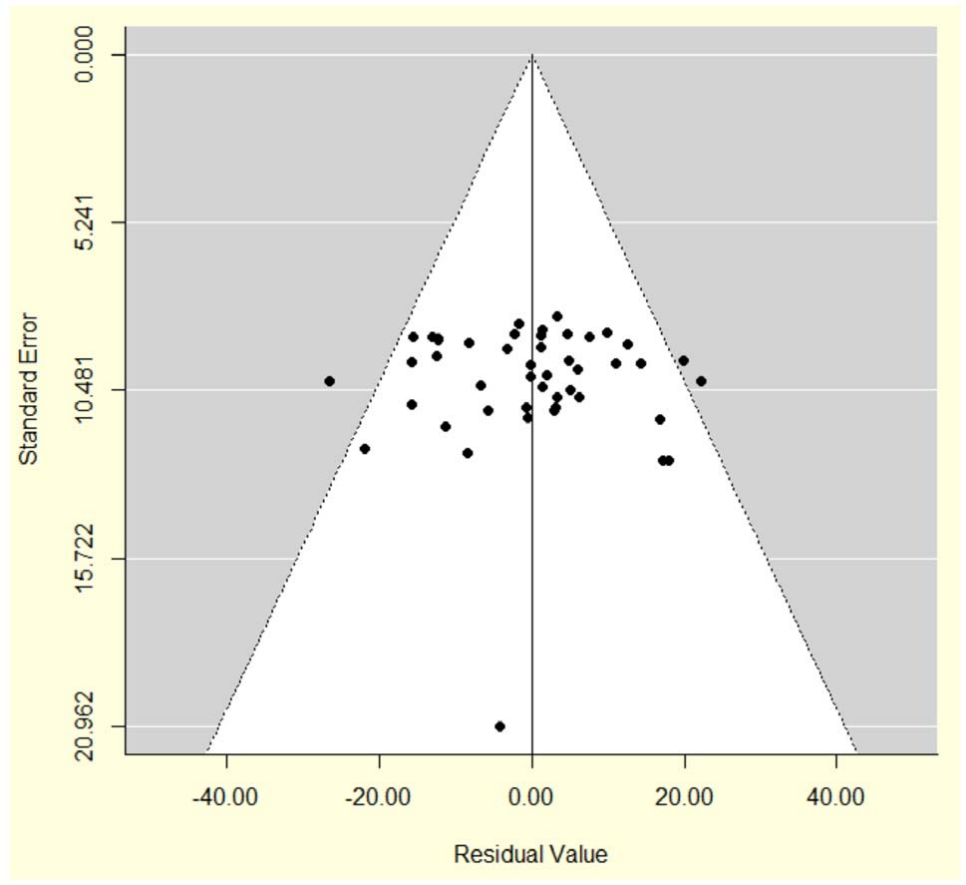
Funnel plot showing a measure of variability (the standard error) of the cancer detection rate against the residuals from the meta-regression model with results shown in Table 3 (main results table). The points represent different studies. Studies are distributed evenly either side of the zero line suggesting no clear evidence of publication bias.

The following data were extracted from each paper: first author, year of publication, study size, mean patient age, mean PSA at repeat biopsy, mean number of previous biopsy episodes, mean number of biopsy cores taken at repeat biopsy, cancer detection rate, and Gleason scores of detected cancers. Statistical analysis was performed by RCH and RAP. For each strategy, weighted summary statistics, which weight according to the sample size of each individual study, were calculated for mean patient age, mean PSA, mean number of previous biopsy episodes, and mean number of cores obtained by the repeat biopsy strategy. We were not able to weight by the inverse study variance for these variables because estimates of within-study variability were unavailable. For the primary outcome (cancer detection rate) however an estimate of the variance could be extracted using the formula for the variance of a proportion, and so this variable was weighted by the inverse variances. A meta-regression analysis compared the overall prostate cancer detection rate (percentage of patients diagnosed with prostate cancer) between the three re-biopsy strategies having adjusted for differences in mean patient age and PSA between the strategy cohorts. In order to account for heterogeneity between studies, a random-effects meta-regression analysis was conducted using inverse-variance weights. The amount of residual heterogeneity between studies was assessed using a Q-test, based on the DerSimonian-Laird estimator. Publication bias was assessed using a funnel plot of the inverse sample variance against model residuals (Figure 1) and a rank correlation test for funnel plot asymmetry performed [24]. A sensitivity analysis was then performed to see if adjusting for the number of previous biopsy episodes affected the meta-regression results. Finally, to compare average pathological outcomes across the repeat biopsy strategies, a Fisher’s Exact test was applied to test for any association between average tumour grade and re-biopsy strategy. The ‘metafor’ package [25] in R software [26] was used to perform the meta-regression analysis. R software was also used to produce the bubble plot and perform the Fisher’s Exact test for the Gleason score analysis. SPSS software was used for all other analyses (SPSS/PASW for Windows, Rel. 18.0.3. 2010. Chicago: SPSS Inc.).

## Results

### Data Analysed

**Figure 2:**
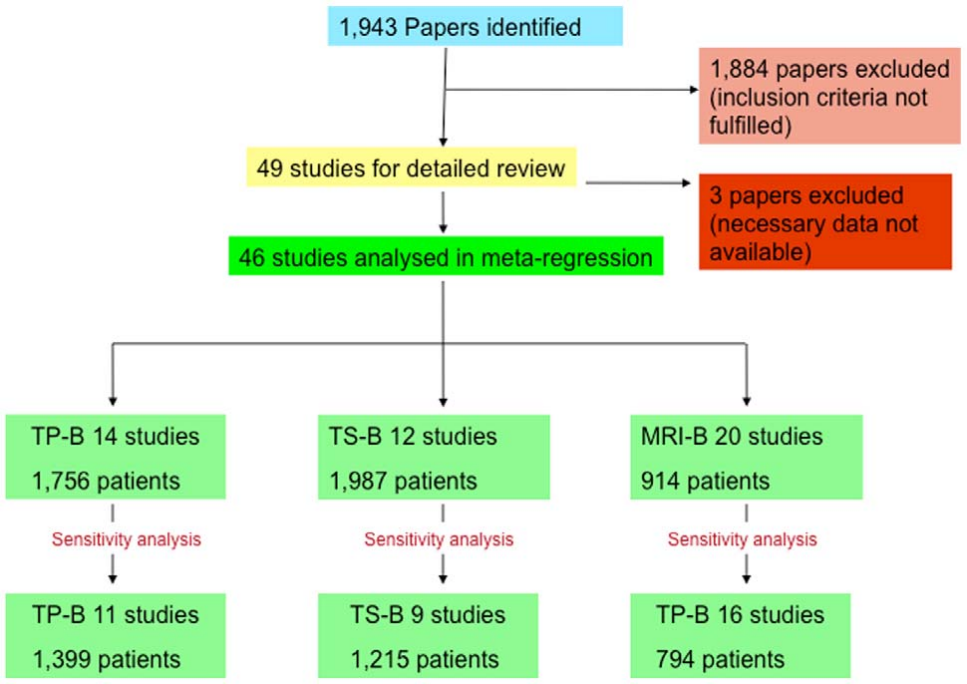
Literature search results (TP-B- Transperineal biopsy, TS-B-Transrectal saturation biopsy, MRI-B – MRI guided biopsy).

The literature search yielded 1,943 papers, which were then individually screened for this study. 1,884 papers were excluded, as they did not meet the inclusion criteria. Forty-nine papers met the inclusion criteria for this study, however three of these were subsequently excluded. In two of these papers data on mean age and mean PSA was not available, [27], [28] and in one paper using both transperineal and transrectal biopsy approaches the cancer detection rates with respect to each approach were not reported separately [29]. Therefore, the necessary data for analysis could not be extracted from the published report. A final 46 papers were included in this analysis comprising 14 studies of TP-B, [23], [30]–[42] 12 studies of TS-B, [30], [43]–[53] and 20 studies of MRI-B [12]–[18], [20], [54]–[65] (Figure 2, PRISMA flow diagram Figure S1, PRISMA checklist Figure S2). The total number of patients included in these papers was 4,657. A funnel plot of the model residuals against standard error showed no clear evidence of publication bias (Figure 1) and the rank correlation test of residuals against sampling variance produced a low Kendall’s tau correlation coefficient of 0·06 (p = 0·56), demonstrating no clear evidence of publication bias.

**Figure 3:**
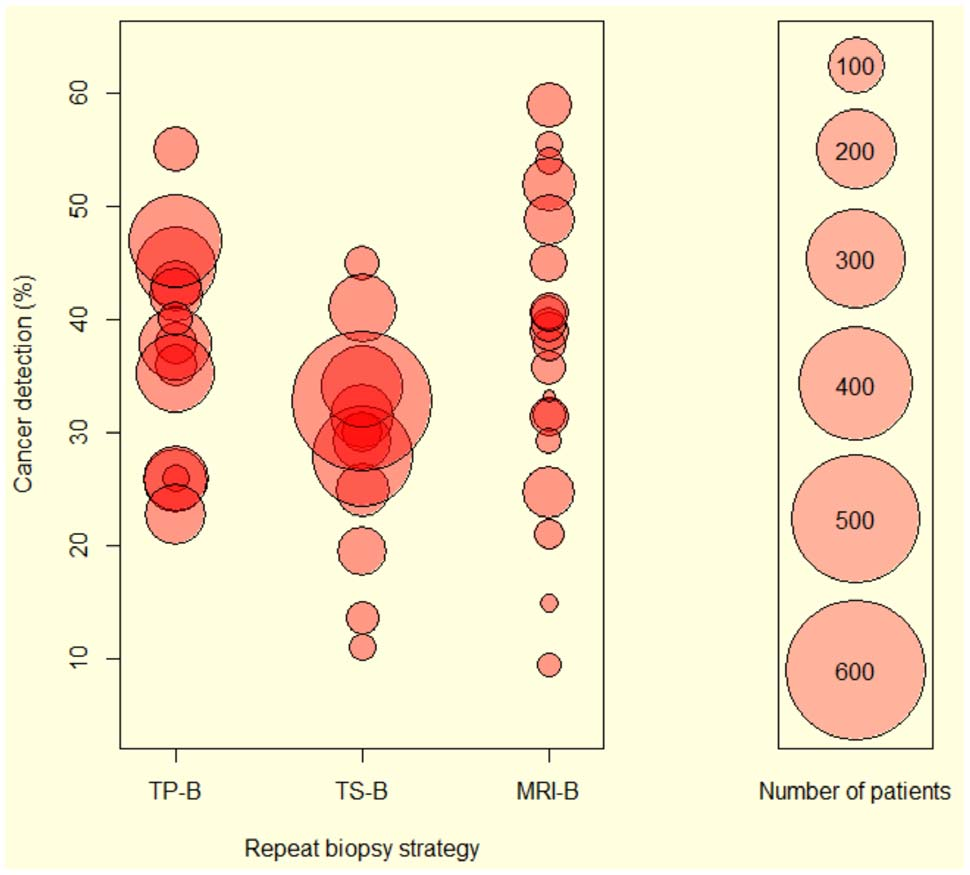
Bubble plot showing the cancer detection rates and respective size of each study in the three strategies. The size of the bubble corresponds to the number of patients included (chart guide on the right of the figure). TP-B- Transperineal biopsy, TS-B-Transrectal saturation biopsy, MRI-B – MRI guided biopsy.

Baseline characteristics of the studies analysed are shown in Table 1. The cohort size of each study and the cancer detection rates for the three re-biopsy strategies are represented as a bubble plot in Figure 3. Overall the TS-B approach had the largest population sizes while MRI-B studies tended to include the smallest numbers of patients. Of the 20 MRI-B studies analysed, 18 obtained prostate biopsies by the transrectal route, one by the transperineal route, and one by the transgluteal route. Four studies used T1 and T2 weighted MRI, seven used T1 and T2 weighted MRI with MRSI, four used T1 and T2 weighted MRI with DCE- MRI, one used T1 and T2 weighted MRI with MRI-DW, two used T1 and T2 weighted MRI, MRSI and DCE-MRI, and two used T1 and T2 weighted MRI, MRSI, DCE-MRI and MRI-DW.

**Table 1:** Baseline characteristics of studies analysed in the meta-regression.

Paper	Year	Number of patients	Strategy	Mean Age/Years (Range)	Mean PSA ng/dL (Range)	Number of previous biopsy sessions(mean) [median]	Mean numberof cores	Gleason Median (Range)	Cancer detection rate (%)
**Abdollah** [30]	2011	140	TP-B	66·4 (52–79)	10·0 (0·9–31·5)	[1·0]	24·0	n/a (n/a)	25·7
**Bittner** [31]	2009	217	TP-B	64·2 (45–83)	8·5 (1·6–96·3)	(1·8)	53·8	6 (6–9)	44·7
**Bott** [32]	2006	60	TP-B	64·0 (n/a)	12·9 (4·6–35·7)	[2·0]	24·0	6 (6–9)	38·0
**Demura** [33]	2005	59	TP-B	64·4 (n/a)	18·0 (n/a)	n/a	20·8	n/a (n/a)	36·0
**Dimmen** [23]	2011	69	TP-B	62·8 (42–78)	19·8 (4·2–229)	(2·4)	19·9	7 (5–9)	55·0
**Furuno** [34]	2004	27	TP-B	64·0 (53–75)	6·9 (4·6–9·8)	(1·4)	19·0	n/a (n/a)	26·0
**Igel** [35]	2001	88	TP-B	65·0 (54–79)	13·1 (n/a)	n/a	17·0	6 (4–9)	43·0
**Merrick** [36]	2007	101	TP-B	64·8 (n/a)	9·1 (n/a)	(2·1) [2·0]	51·1	7 (6–9)	42·2
**Moran** [37]	2006	180	TP-B	63·1 (44–81)	9·3 (0·8–40·1)	(1·8) [2·0]	41·3	n/a (n/a)	37·8
**Novara** [38]	2010	143	TP-B	66·5 (n/a)	9·0 (n/a)	(1·6)	24·0	6 (n/a)	26·0
**Pal** [39]	2011	40	TP-B	62·9 (49–73)	21·9 (4·7–119)	(2·3) [2·0]	36·0	6 (6–9)	40·0
**Pinkstaff** [40]	2005	210	TP-B	66·3 (46–81)	13·6 (n/a)	n/a	21·2	6 (6–9)	35·2
**Satoh** [41]	2005	128	TP-B	67·0 (37–85)	10·4 (2·4–170)	[1·0]	22·0	n/a (n/a)	22·7
**Taira** [42]	2010	294	TP-B	64·1 (n/a)	9·0 (n/a)	[1·0]	25·7	6 (6–10)	46·9
**Abdollah** [30]	2011	140	TS-B	66·2 (47–82)	9·7 (2·1–26·2)	[2·0]	24·0	n/a (n/a)	31·4
**Borboroglu** [43]	2000	57	TS-B	61·4 (47–72)	8·6 (n/a)	(2·1)	22·5	n/a (6–8)	30·0
**Fleshner** [44]	2002	37	TS-B	62·4 (39–75)	22·4 (7·8–73·8)	(4·24) [4·0]	n/a	7 (6–8)	13·7
**Patel** [45]	2004	100	TS-B	62·1 (n/a)	9·4 (n/a)	(1·7)	24·0	6 (5–9)	25·0
**Rabets** [46]	2004	116	TS-B	62·0 (47–83)	9·2 (1·7–48·6)	(1·7) [1·0]	22·8	6 (4–9)	29·3
**Sajadi** [47]	2007	82	TS-B	61·0 (43–76)	9·1 (1·0–34)	n/a	n/a	6 (6–9)	19·5
**Scattoni** [48]	2011	340	TS-B	64·9 (n/a)	9·1 (n/a)	(1·0)	24·0	n/a (n/a)	27·9
**Simon** [49]	2008	40	TS-B	63·0 (48–72)	12·2 (4·9–68·8)	[2·0]	64·0	n/a (n/a)	45·0
**Stav** [50]	2008	27	TS-B	62·1 (50–74)	19·4 (10·1–49)	n/a	61·7	6 (n/a)	11·1
**Stewart** [51]	2001	224	TS-B	64·2 (44–81)	8·7 (n/a)	(1·8)	23·0	6 (4–10)	34·0
**Walz** [53]	2006	161	TS-B	63·7 (43–84)	13·5 (3·3–125·7)	(2·5) [3·0]	24·2	n/a (n/a)	41·0
**Zaytoun** [52]	2011	663	TS-B	64·0 (41–81)	6·4 (0·4–19·4)	n/a	20·7	n/a (n/a)	32·7
**Amsellem-Ouazana** [16]	2005	42	MRI-B	62·3 (54–74)	12·3 (3·9–35)	(2·04)	10·6	n/a (5–9)	35·7
**Anastasiadis** [54]	2006	27	MRI-B	64·5 (53–76)	11·2 (0·02–32·2)	n/a	5·2	6 (4–9)	55·5
**Bhatia** [55]	2007	21	MRI-B	61·4 (50–77)	13·1 (4·3–46·6)	[1]	13·8	6 (n/a)	9·5
**Beyersdorff** [12]	2002	38	MRI-B	64·6 (46–76)	13·9 (4·0–53·0)	n/a	7·7	n/a (n/a)	31·6
**Cheikh** [56]	2009	93	MRI-B	63·2 (52–74)	9·6 (1·6–40·0)	(1·9)	15·8	6 (5–9)	24·7
**Cirillo** [57]	2008	54	MRI-B	65·4 (n/a)	10·8 (n/a)	[1·0]	n/a	6 (4–8)	31·5
**Engelhard** [58]	2006	37	MRI-B	66·0 (46–75)	10·8 (4·0–48·0)	(1·4) [1·0]	n/a	5 (3–7)	37·8
**Franiel** [17]	2011	54	MRI-B	68·0 (49–78)	12·4 (3·3–65·2)	[2·0]	4·0	6 (6–10)	39·0
**Hadaschik** [59]	2011	49	MRI-B	66·0 (47–78)	8·4 (0·5–49·0)	n/a	23·7	n/a (n/a)	44·9
**Hambrock** [60]	2010	68	MRI-B	63·0 (48–74)	13·0 (4·0–243·0)	[3·0]	4·0	6 (5–9)	59·0
**Lattouf** [15]	2007	26	MRI-B	62·0 (32–76)	8·4 (2·1–85·9)	[3·0]	n/a	6.5 (5–9)	54·0
**Park** [13]	2008	43	MRI-B	62·0 (40–80)	12·0 (2·6–66·4)	(2·0)	n/a	7 (6–9)	39·5
**Perrotti** [61]	1999	33	MRI-B	63·0 (45–75)	11·7 (4·8–36·0)	(2·6) [2·0]	n/a	n/a (n/a)	21·1
**Prando** [62]	2005	42	MRI-B	63·3 (45–75)	6·8 (4·1–15·3)	(3·5) [3·0]	12·0	n/a (n/a)	40·5
**Roethke** [18]	2012	100	MRI-B	64·9 (48–81)	12·3 (3·9–65·0)	[2·0]	4·0	7 (5–9)	52·0
**Sciarra** [63]	2010	90	MRI-B	63·5 (n/a)	6·2 (4·0–9·3)	(1·0) [1·0]	n/a	n/a (n/a)	48·9
**Singh** [64]	2008	13	MRI-B	61·0 (37–74)	4·9 (1·3–12·3)	[1]	10	7 (6–8)	15·0
**Testa** [65]	2010	54	MRI-B	63·9 (52–76)	11·4 (3·0–42·0)	(1·7) [2·0]	n/a	6 (n/a)	40·7
**Yuen** [14]	2004	24	MRI-B	64·5 (58–69)	11·7 (6·1–31·8)	(1·2) [1·0]	12·3	n/a (n/a)	29·2
**Wetter** [20]	2005	6	MRI-B	63·8 (50–73)	9·8 (6·0–20·0)	n/a	n/a	6·5 (6–7)	33·3

(TP-B – Transperineal biopsy, TS-B – Transrectal saturation biopsy, MRI-B – MRI guided biopsy, n/a: data not available).

### Weighted Summary Statistics

The weighted summary values for mean patient age, mean PSA at the time of re-biopsy, mean number of previous biopsy episodes, and mean number of prostate cores obtained at re-biopsy for each strategy are displayed in Table 2. As data relating to within-study variability was unavailable, formal statistical comparison of the summary values was not possible. However, it can be seen that patient mean age, mean PSA and mean number of previous biopsy episodes are similar between the three re-biopsy strategies. Cancer detection rates were estimated to be 28·4% (95% CI 22·0 to 34·7%), 37·1% (95% CI 31·7 to 42·5%), and 37·2% (95% CI 30·9 to 43·4%) for TS-B, TP-B and MRI-B respectively. After taking into account the study variances, the cancer detection rates were calculated to be 30·0%, 36·8%, and 37·6% for TS-B, TP-B and MRI-B respectively. The mean number of cores obtained by TP-B and TS-B was greater than MRI-B.

**Table 2:** Weighted summary statistics of data extracted from each paper by repeat biopsy strategy.

Strategy (no. studies) [no. patients]	TP-B (14) [1756]	TS-B (12) [1987]	MRI-B (20) [914]	Overall (46) [4,657]
**Mean Age (years)**	64·9	63·8	64·0	64·3
**Mean PSA (ng/L)**	11·0	9·0	10·6	10·1
**Mean No. previous biopsy episodes** ‡	1·5	1·8	1·9	1·7
**Mean No. Cores at repeat biopsy**	30·4	24·0	9·8	24·8
**Cancer Detection Rate (%)**	36·8	30·0	37·6	34·0

‡ For the studies that do not report the mean number of biopsies, the median was used instead where possible. (TP-B – Transperineal biopsy, TS – B, Transrectal saturation biopsy, MRI-B – MRI guided biopsy).

### Meta-regression Analysis of Cancer Detection Rates

Forty-six studies comprising 14 TP-B, 12 TS-B, and 20 MRI-B papers were analysed. In the meta-regression analysis MRI-B had a significantly higher cancer detection rate than TS-B (8·55%, 95% CI 0·94 to 16·17, p = 0·03) (Table 3). However, there was no statistically significant difference in the cancer detection rate between TP-B and TS-B (7·91%, 95% −0·44 to 16·26, p = 0·06) or between MRI-B and TP-B (0·64%, 95% CI −6·97 to 8·25, p = 0·87). Mean age and PSA at re-biopsy were not significant covariates in this model. Thus following adjustment for mean age and PSA, the strategy used was the only variable which significantly affected the cancer detection rate. After accounting for differences due to strategy, mean age and mean PSA in the meta-regression analysis, the amount of residual heterogeneity between studies was calculated to be 76.0, which is highly significant at the 5% level (QE = 176.8, p<0.0001).

**Table 3:** Meta-regression analysis with cancer detection rate of each strategy as the primary outcome.

		Unstandardised Coefficients	95% Confidence Interval. Lower bound	95% ConfidenceInterval. Upper bound	P-value
**Variables**	**TS-B** (Reference)				
	**TP-B**	7·91	−0·44	16·26	0·063
	**MRI-B**	8·55	0·94	16·17	0·028
	**Mean Age**	0·46	−1·55	2·47	0·653
	**Mean PSA**	−0·13	−0·96	0·70	0·764

TS-B is shown as the reference category for the purpose of comparison with the other two strategies. (TP-B – Transperineal biopsy, TS-B – Transrectal saturation biopsy, MRI-B – MRI guided biopsy).

A sensitivity analysis was next performed, adjusting for the mean number of biopsy episodes prior to the re-biopsy strategy (Table 4). Ten studies were excluded from this analysis due to missing data. Thus a total of 36 studies were eligible for use in this sub-analysis (11 TP-B, [23], [30]–[32], [34], [36]–[39], [41], [42] nine TS-B, [30], [43]–[46], [48]–[50], [53] and 16 MRI-B papers [13]–[18], [55]–[58], [60]–[65]). Again the test for residual heterogeneity was highly significant (QE = 142.7, p<0.0001). Here however we were no longer able to identify any significant difference between the three strategies (TP-B versus TS-B p = 0·12, MRI-B versus TS-B p = 0·16, MRI-B versus TP-B p = 0·74) (Table 4). The most likely reason for this was the reduced statistical power resulting from the smaller sample size, because after re-fitting the original model to the subset of 36 studies, the results were also non-significant (TP-B versus TS-B 6·29%, 95% CI −3·57 to 16·15, p = 0·21); MRI-B versus TS-B 5·98%, 95% CI −3·13 to 15·10, p = 0·20; MRI-B versus TP-B, −0·31%, 95% CI −9·29 to 8·67, p = 0·95).

**Table 4:** Meta-regression analysis with cancer detection rate of each strategy as the primary outcome, adjusting for mean age, mean PSA, and mean number of previous biopsies.

		Unstandardised Coefficients	95% Confidence Interval. Lower bound	95% Confidence Interval.Upper bound	P-value
**Variables**	**TS-B** (Reference)				
	**TP-B**	7·99	−2·04	18·02	0·118
	**MRI-B**	6·47	−2·63	15·57	0·164
	**Mean Age**	0·36	−2·03	2·75	0·768
	**Mean PSA**	−0·36	−1·55	0·84	0·558
	**Mean number of previous biopsies**	5·23	−1·00	11·45	0·100

TS-B is shown as the reference category for the purpose of comparison with the other two strategies. (TP-B – Transperineal biopsy, TS-B –Transrectal saturation biopsy, MRI-B – MRI guided biopsy).

### Analysis of Pathological Grades

Twenty-eight studies (61%) had median Gleason score available for this analysis. Comparison of median Gleason scores by Fisher’s exact test did not reveal any significant differences between the strategies (p = 0·90). As only 28 studies had complete data available for analysis of Gleason score we interpret these results with caution. Nevertheless, we were unable to find any evidence of a difference in the reported clinical significance (as defined by Gleason sum) of the detected cancers between strategies.

## Discussion

In this study we addressed the question of which repeat biopsy strategy is most effective at diagnosing prostate cancer in men with initial negative TRUS-B and ongoing suspicion of prostate cancer. There is currently no published consensus on which re-biopsy approach should be used in this group of men and the decision as to which strategy to use is largely based on institutional practice and the availability of a particular technology. A key observation was the large heterogeneity across the studies included in the analysis with regards the patient demographics, biopsies taken, previous biopsy episodes, and outcome reporting. Our conclusions were therefore based on analyses following correction for these variables as much as possible. In the initial meta-regression analysis our results demonstrated that MRI-B detected significantly more cancer than TS-B, but there were no significant differences between either MRI-B and TP-B, or TP-B and TS-B. In a subset analysis correcting for number of prior biopsy episodes however, this difference was not maintained. This was almost certainly due to a loss of statistical power resulting from the reduced number of studies available for inclusion in the sensitivity analysis as when the 36 studies from the sensitivity analysis were re-analysed using the initial meta-regression model, there were no significant differences between the three re-biopsy strategies. Additionally, the weighted summary statistics (Table 2) demonstrated a similar mean number of previous biopsy episodes between the three strategies, therefore it is unlikely that the inclusion of this variable would influence the meta-regression results. However, our analysis was not able to conclusively demonstrate a clear benefit of one approach over another in cancer detection rates. These results therefore suggest that at present no one re-biopsy method can be recommended based solely on current evidence available from the literature.

In this study TP-B and MRI-B had very similar cancer detection rates but the latter achieved this with fewer biopsy cores. It could therefore be said that MRI-B offers higher cancer yield per biopsy core than the other strategies. These results are consistent with the recent systematic analysis by Moore [22] although that study did not differentiate between initial and re-biopsy patients. It was not possible to formally compare complication rates between the three re-biopsy strategies as part of this analysis due to a lack of reported data in many studies. Previous studies have not found a correlation between number of cores and complication rates [66] and so further research is needed to establish the complications associated with each of the re-biopsy strategies analysed here.

It is well recognised that many detected cancers will be indolent and not require active intervention [67]. The ideal biopsy strategy therefore would achieve the maximal detection of clinically significant cancers with fewest biopsy cores. In this regard MRI-B may have the advantage as it alone allows a guided biopsy based on a priori radiological knowledge of the prostate architecture and morphology [22]. MRI-B however does require significantly greater multidisciplinary input in its planning and execution as well as access to good quality imaging and reporting. This may not be available in all centres though the use of functional MRI in routine clinical practice is expanding [68]. Certainly our data would suggest that the cancer yield of TP-B does not significantly differ from MRI-B, and would therefore be a reasonable alternative to MRI-B.

The use of MRI for targeted prostate biopsy is a rapidly evolving field and recent data suggests an improving ability to better detect clinically significant cancers after initial negative investigation [22], [69], [70]. In this analysis we were only able to assess for differences in Gleason sum score between the groups and found no significant difference in median scores. Clinical significance of a tumour however may also be derived from other variables such as the extent of core involvement and number of cores involved. At the present time, there is no consensus on how best to perform MRI-B in terms of both the approach to the prostate and the choice of imaging modality [22], [71]. The majority of MRI-B studies in this analysis used the transrectal route to obtain prostate biopsies with two studies using the transperineal [59] or transgluteal [20] approach. The use of MRI/transrectal ultrasound fusion to obtain targeted biopsies is also undergoing investigation [59], [64]. In a recent report of 49 patients undergoing repeat biopsy Hadaschik [59] detected cancer in 45% of men using MRI guided transperineal biopsy. Whilst this result is of interest, the sample size is small and there is therefore a need for further comparative evaluation of transperineal MRI-B, to see whether cancer detection in the re-biopsy setting could be further increased by fusion technology. It is clear that imaging guided biopsies will continue to evolve and be focused on detecting clinically significant cancers. Given the likely increased clinical resource needs for MRI-B there is a clear rationale for well designed prospective studies to compare the outcomes of different biopsy approaches. This ideally should be in a randomised setting and, if not feasible, then by robust prospective databases incorporating standardised data collection as well as clinical and pathological outcomes reporting. A forthcoming UK Health Technology Assessment report due to be published this year, on the diagnostic accuracy and cost-effectiveness of MRI techniques in prostate biopsy may provide further information in this area. Assessment of the diagnostic efficacy of any re-biopsy strategy should, ideally be based on correlation of the biopsy findings with step-sectioned radical prostatectomy specimens.

This study has a number of inherent limitations. Data was extracted from published manuscripts, rather than from original patient data, so a degree of reporting bias is inevitable. We have acknowledged that there is heterogeneity of data across the studies analysed despite efforts to standardise the data included in the meta-regression analysis. We were unable to formally compare the weighted summary statistics with statistical methodology as data relating to within-study variability was not available from the published manuscripts. We did not differentiate between the various techniques of MRI-B, and so our findings with respect to this strategy may not be applicable to each of the individual techniques described in the literature. Finally, this study was not able to accurately assess and compare the complication rates or costs of each modality as these factors were often not well recorded in the papers reviewed. An evaluation of these aspects in a prospective study will be crucial in determining the health economic cost benefits of each modality.

### Conclusions

This study has compared the cancer detection rates of three re-biopsy strategies in men with initial negative biopsies and ongoing suspicion of prostate cancer. The main meta-regression analysis demonstrated that MRI-B had a significantly higher cancer detection rate than TS-B, but this result was not maintained after adjustment for the mean number of prior biopsy episodes. Notably MRI-B required the fewest biopsy cores to achieve the greatest cancer detection rate. We observed considerable heterogeneity in the studies in the literature. There is an urgent need for prospective national and international audit and a common reporting format to improve the quality of data for accurate comparison of biopsy strategies and to investigate the complication rates associated with each strategy. Quality of life and health economic analyses should also be incorporated to better inform clinical decision on the most efficient and cost effective re-biopsy strategy.

## Supporting Information

Figure S1 **Details of the literature search strategy and results are displayed in the PRISMA flow diagram.**
(DOC)

Figure S2 **The PRISMA checklist, outlining the accepted standard for meta-analysis.**
(PDF)
