# Supplementary material for: Repeat Prostate Biopsy Strategies after Initial Negative Biopsy: Meta-Regression Comparing Cancer Detection of Transperineal, Transrectal Saturation and MRI Guided Biopsy
Source: PLoS One. 2013 Feb 27;8(2):e57480. doi: 10.1371/journal.pone.0057480 (PMC3583836; doi:10.1371/journal.pone.0057480)
Supplement: Figure S1 — Details of the literature search strategy and results are displayed in the PRISMA flow diagram. (DOC) [file pone.0057480.s001.doc]

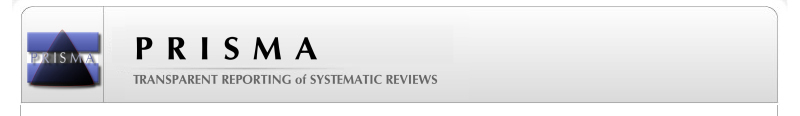
**PRISMA 2009 Flow Diagram**

**Screening**

**Included**

**Eligibility**

**Identification**

Records identified through database searching
(n = 1,943)

Additional records identified through other sources
(n = 0 )

Records after duplicates removed
(n = 1,943)

Records screened
(n = 1,943)

Records excluded
(n = 1,884)

Full-text articles assessed for eligibility
(n = 49)

Full-text articles excluded,
(n = 3) Necessary data not available for extraction

Studies included in qualitative synthesis
(n = 46)

Studies included in quantitative synthesis (meta-analysis)
(n = 46)
